# Hybrid optimization method with general switching strategy for parameter estimation

**DOI:** 10.1186/1752-0509-2-26

**Published:** 2008-03-24

**Authors:** Eva Balsa-Canto, Martin Peifer, Julio R Banga, Jens Timmer, Christian Fleck

**Affiliations:** 1 Institute of Physics, University of Freiburg, Germany; 2 Freiburg Centre for Systems Biology, Germany; 3 Process Engineering Group, Spanish Council for Scientific Research, IIM-CSIC, Spain

## Abstract

**Background:**

Modeling and simulation of cellular signaling and metabolic pathways as networks of biochemical reactions yields sets of non-linear ordinary differential equations. These models usually depend on several parameters and initial conditions. If these parameters are unknown, results from simulation studies can be misleading. Such a scenario can be avoided by fitting the model to experimental data before analyzing the system. This involves parameter estimation which is usually performed by minimizing a cost function which quantifies the difference between model predictions and measurements. Mathematically, this is formulated as a non-linear optimization problem which often results to be multi-modal (non-convex), rendering local optimization methods detrimental.

**Results:**

In this work we propose a new hybrid global method, based on the combination of an evolutionary search strategy with a local multiple-shooting approach, which offers a reliable and efficient alternative for the solution of large scale parameter estimation problems.

**Conclusion:**

The presented new hybrid strategy offers two main advantages over previous approaches: First, it is equipped with a switching strategy which allows the systematic determination of the transition from the local to global search. This avoids computationally expensive tests in advance. Second, using multiple-shooting as the local search procedure reduces the multi-modality of the non-linear optimization problem significantly. Because multiple-shooting avoids possible spurious solutions in the vicinity of the global optimum it often outperforms the frequently used initial value approach (single-shooting). Thereby, the use of multiple-shooting yields an enhanced robustness of the hybrid approach.

## Background

The goal of systems biology is to shed light onto the functionality of living cells and how they can be influenced to achieve a certain behavior. Systems Biology therefore aims to provide a holistic view of the interaction and the dynamical relation between various intracellular biochemical pathways. Often, such pathways are qualitatively known which serves as a starting point for deriving a mathematical model. In these models, however, most of the parameters are generally unknown, which thus hampers the possibility for performing quantitative predictions. Modern experimental techniques can be used to obtain time-series data of the biological system under consideration from which unknown parameters values can be estimated. Since these data are often sparsely sampled, parameter estimation is still an important challenge in these systems. On the other hand, the use of model-based (*in silico*) experimentation can greatly reduce the effort and cost of biological experiments, and simultaneously facilitates the understanding of complex biological systems. In particular, the modeling and simulation of cellular signaling pathways as networks of biochemical reactions has recently received major attention [1]. These models depend on several parameters such as kinetic constants or molecular diffusion constants which are in many cases not accessible to experimental determination. Therefore, it is necessary to solve the so-called inverse problem which consists of estimating unknown parameters by fitting the model to experimental data, i.e., by solving the model calibration or parameter estimation problem.

Parameter estimation is usually performed by minimizing a cost function which quantifies the differences between model predictions and measured data. In general, this is mathematically formulated as a non-linear optimization problem which often results to be multi-modal (non-convex). Most of the currently available optimization algorithms, specially local deterministic methods, may lead to suboptimal solutions if multiple local optima are present, as shown in [2,3]. This is particularly important in the case of parameter estimation for biological systems, since in most cases no clear intuition even about the order of magnitude exists. Finding the correct solution (global optimum) of the model calibration problem is thus an integral part of the analysis of dynamic biological systems. Consequently, there has been a growing interest in developing procedures which attempt to locate the global optimum. In this concern, the use of deterministic [4-9] and stochastic global optimization methods [10-12] have been suggested. For deterministic global optimization routines the convergence to the global optimum is guaranteed but this approach is only feasible for a considerably small number of parameters. Stochastic global optimizers on the other side converges rapidly to the vicinity of the global solution, although further refinements are typically costly. In other words, finding the location of the optimum is computationally expensive, especially for large systems as found in systems biology. Alternatively, Rodriguez-Fernandez et al. [2] propose a hybrid method to exploit the advantages of combining global with local strategies. That is, robustness in finding the vicinity of the solution using the global optimization procedure and the fast convergence to solution by the local optimization procedure. At a certain point the search is switched from using the global optimizer to the local optimization routine by this hybrid strategy. The determination of the so called switching point is done on the basis of exhaustive numerical simulations prior to the actual optimization run.

In this work a refined hybrid strategy is proposed which offers two main advantages over previous alternatives [2]: First, we employ a multiple-shooting method which enhances the stability of the local search strategy. Second, we propose a systematic and robust determination of the switching point. Since the calculation of the switching point can be done during the parameter estimation itself, computationally expensive simulations are no longer needed.

### Parameter estimation in dynamical systems

Generally, the parameter estimation problem can be stated as follows. Suppose that a dynamical system is given by the *d*-dimensional state variable *x*(*t*) ∈ ℝ^*d *^at time *t *∈ *I *= [*t*_0_, *t*_*f*_], which is the unique and differentiable solution of the initial value problem

(1) $$\begin{array}{cc} \dot{x}(t)=f(x(t),t,p) & x(t_{0})=x_{0}. \end{array}$$

The right-hand side of the ODE depends in addition on some parameters $p\in {\mathbb{R}}^{n_{p}}$. It is further assumed that *f *is continuously differentiable with respect to the state *x *and parameters *p*. Let *Y*_*ij *_denote the data of measurement *i *= 1, ..., *n *and of observable *j *= 1, ..., *N*, whereas *n *represents the total amount of data and *N *is the number of observables. Moreover, the data *Y*_*ij *_satisfies the observation equation

(2) *Y*_*ij *_= *g*_*j*_(*x*(*t*_*i*_), *p*) + *σ*_*ij*_ε_*ij *_    *i *= 1,...,*n*,

for some observation function *g *: ℝ^*d *^→ ℝ^*N*^, *d *≥ *N*, *σ*_*ij *_> 0, where *ε*_*i*_'s are independent and standard Gaussian distributed random variables. The sample points *t*_*i *_are ordered such that *t*_0 _≤ *t*_1 _< ...; <*t*_*n *_≤ *t*_*f *_and the observation function *g *is again continuously differentiable in both variables. Eqs. (1) and (2) define an single-experiment design. If several experiments are available, possibly under different experimental conditions, Eq. (2) depends on each experiment and must be modified in the following manner

(3) *Y*_*ijk *_= *g*_*j*_(*x*(*t*_*i*_), *p*) + *σ*_*ijk*_*ε*_*ijk *_    *k *= 1, ..., *n*_*exp*_.

Certain parameters may be different for each experiment, but the treatment of these local parameters and the different experiments requires only obvious modifications of the described procedures and therefore only the single-experiment design *n*_*exp *_= 1 is discussed in the following for sake of clarity.

On the basis of the measurements (*Y*_*i*_)_*i *= 1,...,*n *_the task is now to estimate the initial state *x*_0 _and the parameters *p*. The principle of maximum-likelihood yields an appropriate cost function which has to be minimized with respect to the decision variables *x*_0 _and *p*. Defining *x*(*t*_*i*_; *x*_0_, *p*) as being the trajectory at time *t*_*i*_, the cost function is then given by

(4) $$ℒ(x_{0},p)=\sum_{i=1}^{n}\sum_{j=1}^{N}\frac{{\left(Y_{ij}-g_{j}(x(t_{i};x_{0},p),p)\right)}^{2}}{2\sigma_{ij}^{2}}.$$

In general, minimizing ℒ is a formidable task, which requires advanced numerical techniques.

## Methods

Mathematical modeling in systems biology rely on quantitative information of biological components and their reaction kinetics. Due to paucity of quantitative data, various numerical optimization techniques have been employed to estimate parameters of such biological systems. Employed optimization techniques include local, deterministic approaches like Levenberg-Marquardt algorithm, Sequential Quadratic Programming, and stochastic approaches like Simulated Annealing, Genetic Algorithms and Evolutionary Algorithms (see for example, [10,13]). Most commonly, local methods optimize the cost function, Eq. (4), directly with respect to initial values *x*_0 _and parameters *p*. This optimization scheme is called initial value approach or alternatively single-shooting. Huge differences in the performance can be observed if either local or global optimization methods are used. Due to the presence of multiple minima in Eq. (4), convergence of local optimization methods to the global minimum is in most cases limited to a rather small domain in search space, see, e.g., [2,3]. In contrast, global methods have generally a substantially larger convergence domain but the computational cost increases drastically.

One of the simplest global methods is a multistart method. Here, a large amount of initial guesses are drawn from a distribution and subjected to a parameter estimation algorithm based on a local optimization approach. The smallest minimum is then regarded as being the global optimum. In practice, however, there is no guarantee of arriving to the global solution and the computational effort can be quite large. These difficulties are arising because it is a-priori not clear how many random initial guesses are necessary. Over the last decade more suitable techniques for the solution of multi-modal optimization problems have been developed (see, e.g., [14] for a review). Several recent works propose the application of global deterministic methods for model calibration in the context of chemical processes, biochemical processes, metabolic pathways, and signaling pathways [4-6,8,9]. Global deterministic methods in general take advantage of the problem's structure and even guarantee convergence within a preselected level of accuracy. Although very promising and powerful, there are still limitations to their application, manly due to rapid increase of computational cost with the size of the considered system and the number of its parameters. As opposed to deterministic approaches, global stochastic methods do not require any assumptions about the problem's structure. Stochastic global optimization algorithms are making use of pseudo-random sequences to determine search directions toward the global optimum. This leads to an increasing probability of finding the global optimum during the runtime of the algorithm. The main advantage of these methods is that they rapidly arrive to the proximity of the solution. Examples of global stochastic methods are: pure random search algorithms, evolutionary strategies, genetic algorithms, scatter search and clustering methods. Some of these strategies have been successfully applied to parameter estimation problems in the context of systems biology, see [10,11,15].

In [2] a combination of global stochastic methods with local methods has been proposed. This, so called hybrid approach, utilizes the property of the global search strategy to arrive quickly to the vicinity of the solution. At a certain point in the proximity of the solution the optimizer is switched from the global stochastic to the local deterministic search method. It has been shown that this strategy saves a huge amount of computational effort and provides an efficient and robust alternative for model calibration. Therefore, the hybrid method takes advantage of the complementary strengths of both optimization strategies: global convergence properties in the case of the stochastic method, and fast local convergence in the case of the deterministic approach. Speed and the stability, however, of the resulting hybrid approach also depends on the performance of the used local approach. For this reason we choose the method of multiple-shooting rather than the initial value approach in order to refine the hybrid optimization strategy as described in [2]. As shown below multiple-shooting has in general a larger domain of convergence to the global optimum while only a small portion of additional computational load has to be taken into account compared to single shooting. A brief outline of the multiple-shooting method is given below.

### Multiple-shooting

Detailed discussion and some applications to measured data of the method can be found, e.g., in [16-22]. Here, we will concentrate on the main principles of multiple-shooting in order to construct a new hybrid approach. The basic idea of multiple-shooting is to subdivide the time interval *I *= [*t*_0_, *t*_*f*_] into *n*_*ms *_<*n *subintervals *I*_*k *_such that each interval contains at least one measurement. Each of the intervals are assigned to an individual experiment having its own initial values ${\left(x_{0}^{k}\right)}_{k=1,\cdots ,n_{ms}}$ but sharing the same parameters *p*. Suppose that *x*(*t*_*i*_; $x_{0}^{k}$, *p*) for all *k *= 1, ..., *n*_*ms *_denotes the trajectory within an interval. Since the total trajectory for each *t *∈ *I *= *I*_1 _∪ ... ∪$I_{n_{ms}}$ is usually discontinuous at the joins of the subintervals, smoothness as anticipated by the solution of Eq. (1) is not fulfilled. To enforce smoothness, the optimization is constrained such that all discontinuities are removed at convergence. This leads to a constrained non-linear optimisation problem, which has in addition the advantage that further equality and inequality constraints can easily be implemented. Note that if the integration between two time points is numerically unfeasible, the segment where this problem occurs can be removed. This, however, leads to a split trajectory which parts can be treated using a multiple-experiment fit.

For each *k *= 1, ... *n*_*ms *_let $t_{k}^{+}=\max\{I_{k}\},t_{k}^{-}=\min\{I_{k}\}$ and *θ*_*k *_= ($x_{0}^{k}$, *p*) the optimization problem can then be formulated in the following manner:

(5) $$\begin{array}{ll} & ℒ(\theta_{1},\cdots ,\theta_{n_{ms}})=\frac{1}{2}\sum_{j=1}^{N}\sum_{k=1}^{n_{ms}}\sum_{\left\{i:t_{i}\in I_{k}\right\}}{\left(R_{ijk}^{a}(\theta_{k})\right)}^{2}=\min_{\theta_{1},\cdots ,\theta_{n_{ms}}}\\ \text{subject to} & \\ & \begin{array}{ll} x(t_{i}^{+};\theta_{i})-x(t_{i+1}^{-};\theta_{i+1}) & i=1,\cdots ,n_{ms}-1\\ R_{j}^{e}(\theta_{1},\cdots ,\theta_{n_{ms}})=0 & j=1,\cdots ,n_{e}\\ R_{k}^{g}(\theta_{1},\cdots ,\theta_{n_{ms}})\geq 0 & k=1,\cdots ,n_{g}, \end{array} \end{array}$$

where the continuity constraints are given at the first row of the constraints-part, followed by optional constraints $R_{j}^{e},R_{k}^{g}$. Cost function ℒ(*θ*_1_, ... $\theta_{n_{ms}}$) is equivalent to Eq. (4) if the continuity constraints are satisfied; hence

(6) $$R_{ijk}^{a}(\theta_{k})=\frac{Y_{ij}-g_{j}(x(t_{i};\theta_{k}),p)}{\sigma_{ij}}.$$

We solved the non-linear programming problem defined by Eq. (5) iteratively by employing a generalized-quasi-Newton method [23,24]. With the current guess $\theta^{l-1}=(\theta_{1}^{l-1},\cdots ,\theta_{n_{ms}}^{l-1})$, the update step $\Delta \theta^{l}=(\Delta \theta_{1}^{l},\cdots ,\Delta \theta_{n_{ms}}^{l})$ for the *l*-th iteration is obtained by solving the resulting linearly constrained least squares problem:

(7) $$\begin{array}{ll} & \frac{1}{2}\sum_{j=1}^{N}\sum_{k=1}^{n_{ms}}\sum_{\left\{i:t_{i}\in I_{k}\right\}}{\left(R_{ijk}^{a}\left(\theta_{k}^{l-1}\right)+{\text{d}}_{\theta }R_{ijk}^{a}\left(\theta_{k}^{l-1}\right)\Delta \theta^{l}\right)}^{2}=\min_{\Delta \theta^{l}}\\ \text{subject to} & \\ & \begin{array}{l} x(t_{i}^{+};\theta_{i}^{l-1})-x(t_{i+1}^{-};\theta_{i+1}^{0})+{\text{d}}_{\theta_{i}}x(t_{i}^{+};\theta_{i}^{l-1})\Delta \theta_{i}^{l}-{\text{d}}_{\theta_{i+1}}x(t_{i+1}^{-};\theta_{i+1}^{l-1})\Delta \theta_{i+1}^{l}=0\\ R_{j}^{e}(\theta^{l-1})+{\text{d}}_{\theta }R_{j}^{e}(\theta^{l-1})\Delta \theta^{l}=0\\ R_{k}^{g}(\theta^{l-1})+{\text{d}}_{\theta }R_{k}^{g}(\theta^{l-1})\Delta \theta^{l}\geq 0, \end{array} \end{array}$$

where d_*θ *_denotes the derivative with respect to the parameters *θ *of the corresponding function. Setting *θ*^*l *^= *θ*^*l*-1 ^+ Δ*θ*^*l *^and repeating Eq. (7) until Δ*θ*^*l *^≈ 0, yields the desired parameter estimates under the condition that all parameters itself are identifiable and the constraints are not contradictory. These extra assumptions are necessary to fulfil the so called Kuhn-Tucker conditions for the solvability of constrained, non-linear optimization problems [23,25].

In combination with multiple-shooting the generalized-quasi-Newton approach has three major advantages: first, the optimization is sub-quadratically convergent. Second, a transformation of Eqs. (7) can be found such that the transformed equations are numerically equivalent to the initial value approach. Third, due to the linearization of the continuity constraints, they do not have to be fulfilled exactly after each iteration, but only at convergence. This allows discontinuous trajectories during the optimization process, reducing the problem of local minima. The first two properties yield the desired speed of convergence whereas the third property is mainly responsible for the stability of multiple-shooting. This is gained by the possibility that the algorithm can circumvent local minima by allowing for discontinuous trajectories while searching the global minimum. Whereas, the main disadvantage is due to the linearization of the cost function. It can easily happen that despite the update step Δ*θ*^*l *^is pointing in the direction of decreasing ℒ the proposed step is too large. Such an overshooting is common to any simple optimization procedures based on the local approximation of the cost function. A suitable approach to cure this deficiency is realized by relaxing the update step; hence *θ*^*l *^= *θ*^*l*-1 ^+ λ^*l*^Δ*θ*^*l *^for some λ^*l *^∈ (0, 1]. This procedure is referred to as damping and provides the bases of the determination of the switching point which we propose in the following.

### A new hybrid method

Besides the choice of the global and local optimization procedure, the determination of the switching point is vital for the robustness of the hybrid approach, as discussed in [26]. This is supported by the results presented in [2] where it is shown that different switching points may lead to different solutions and that careful investigations and computationally expensive empirical tests must be consulted in order to determine an appropriate switching strategy. In order to avoid such time consuming tests, we propose a systematic determination of the switching point in the following. All calculations needed to compute the switching point are carried out during the optimization which reduces the computational effort significantly. As global stochastic optimization methods we decide to use evolutionary approaches such as Stochastic Ranking Evolutionary Search (SRES) [27] or Differential Evolution (DE) [28]. The local search method is – as already stated above – multiple-shooting.

#### Calculation of the switching point

The multiple-shooting method is equipped with a relaxation algorithm to prevent overshooting of the update step. This overshooting is due to the quadratic approximation of the likelihood function in Eq. (7) which is often too crude for points far away of the minimum. For these points the calculated update step tends to be too long and might result in a step leading to an increased value of the cost function. The relaxation method, also called damping method, selects some λ^*l *^∈ (0, 1] such that the update step *θ*^*l *^= *θ*^*l*-1 ^+ λ^*l*^Δ*θ*^*l *^is descendant. For this some level function has to be used. Such a level function must share the same monotony properties of the cost function close to the global minimum. Here, the objective to judge whether the proposed step at *θ*^*l*-1 ^is descendant is given by the following level function [17,22,23]:

*T*(λ) = ||*G*(*θ*^*l*-1^)*R*^*a*^(*θ*^*l*-1 ^+ λΔ*θ*^*l*^)||^2^,

where *R*^*a *^is the *n *× *N*-dimensional vector with components $R_{ijk}^{a}$ in Eq. (6) and *G *is the generalized inverse of Eq. (7), satisfying Δ*θ*^*l *^= *G*(*θ*^*l*-1^)*R*^*a*^(*θ*^*l*-1^). Based on *T*(λ) a very efficient corrector-predictor scheme is given in [17,23] to determine the optimal damping parameter λ. Furthermore, it can be shown that whenever the method enters the region of local convergence, the method converges to a full step procedure and thus λ → 1 [17,22,23]. This feature of the damping strategy can be utilized to detect the region of local convergence and provides a suitable criterion for determining the switching point. Calculating λ during the global optimization and successively checking whether λ = 1 yields the desired information about the switching point. For stability reasons we propose to switch to the local method only after a certain number, say *n*_1_, of consecutive λ = 1 is achieved. After the initialization of the method a number of iterations *n*_0 _is performed using the global method without checking the switching point criterion in order to decrease the computational load, note that a minimum of around 15 iterations will be usually needed, this number may be increases if the size of the search space also increases. For the simulations presented in this study *n*_1 _= 2. Since the corrector-predictor scheme can be implemented very efficiently, calculation of the damping parameter λ is computationally inexpensive.

## Results and Discussion

In order to demonstrate the performance of the method we have chosen two examples: the STAT5 signaling pathway [29] and Goodwin's model [30] for a feedback control system showing a Hopf bifurcation. In both cases we simulated data having a noise-to-signal ratio of either 0% or 10% and evaluated the performance of the proposed hybrid method in comparison to local and global search strategies.

### STAT5 signaling pathway

The JAK/STAT (Janus kinase/Signal Transducer and Activator of Transcription) signaling cascade is a well studied pathway stimulating cell proliferation, differentiation, cell migration and apoptosis [31]. A mathematical model of the JAK/STAT5 pathway is, e.g., presented in [29]. Here, the binding of the ligand to the erythropoietin receptor (EpoR) located at the cell membrane results in an activation of the receptor (via cross-phosphorylation of the JAK proteins) and leads to a subsequent phosphorylation of the STAT5 molecule. Two phosphorylated STAT5 proteins form a homodimer which enters the cell nucleus, where it stimulates transcription of target genes. Then the molecules are dedimerized and dephosphorylated and relocated back to the cytoplasm. This process is modeled by the following system of non-linear delay differential equations:

(8) $$\begin{array}{l} {\dot{x}}_{1}=-k_{1}x_{1}EpoR_{A}(t)+k_{2}x_{3}(t-\tau )\\ {\dot{x}}_{2}=-x_{2}^{2}+k_{1}x_{1}EpoR_{A}(t)\\ {\dot{x}}_{3}=-k_{2}x_{3}+x_{2}^{2}\\ {\dot{x}}_{4}=-k_{2}x_{3}(t-\tau )+k_{2}x_{3}, \end{array}$$

where *k*_1_, *k*_2 _are rate constants and *τ *is a delay parameter. The cytoplasmic unphosphorylated STAT5 is represented by *x*_1_, whereas *x*_2 _denotes the phosphorylated STAT5. Moreover, *x*_3 _describes the dimer and *x*_4 _is the nuclear STAT5. The receptor activity is denoted by *EpoR*_*A*_(*t*) and the delay *τ *represents the time the STAT5 proteins reside in the nucleus. Delay differential equation exhibit a rich dynamic, which make them a difficult candidate for parameter estimation [32,33]. We approximate the delay in Eq. (8) by a linear chain of length *N*:

$$\begin{array}{c} {\dot{q}}_{1}=N/\tau \;(in(t)-q_{1})\\ {\dot{q}}_{2}=N/\tau \;(q_{1}-q_{2})\\ \cdots \\ {\dot{q}}_{N-1}=N/\tau \;(q_{N-2}-q_{N-1})\\ out=N/\tau \;(q_{N-1}-out(t)). \end{array}$$

Here, *in*(*t*) is the input and *out*(*t*) the output of the delay chain. We set *in*(*t*) = *x*_3_(*t*), *out*(*t*) = *x*_3_(*t *- *τ*), and *N *= 8. This provides a reasonable approximation of the time delay [32]. Two different sets of data were obtained by numerical simulations with a noise to signal ratio of 0% and 10%, respectively. As observed quantities we choose the total amount of activated STAT5, *y*_1 _= *s*_1_(*x*_2 _+ *x*_3_), and the total amount of STAT5 in the cytoplasm, *y*_2 _= *s*_2_(*x*_1 _+ *x*_2 _+ *x*_3_), where *s*_1 _and *s*_2 _are scaling parameters introduced to deal with the fact that only relative protein amounts are measured. Initial conditions and the kinetic parameters were chosen to be: *x*_1_(0) = 3.71, *x*_*i*_(0) = 0, (*i *= 2,...,4), *k*_1 _= 2.12, *k*_2 _= 0.109, *τ *= 5.2, *s*_1 _= 0.33 and *s*_2 _= 0.26. From the simulated data we aim to estimate the rate constants *k*_1_, *k*_2_, the delay parameter *τ *and the initial concentration of unphosphorylated STAT5 *x*_1_(0). In case of local optimization methods – single and multiple-shooting – we used multistarts, where the initial guess of each restart is randomly chosen from the intervals [0, 5] (Box 5), [0, 10] (Box 10), and [0, 100] (Box 100), respectively, using a uniform distribution. For each box size 100 restarts are chosen. Note that the delay parameter τ has to be restricted to Δ*t *<*τ *< (*t*_*f *_- *t*_0_), where Δ*t *denotes the sampling rate of the data. This follows from the fact that no information is contained in the data about delays smaller than τ*t *and larger than the total measurement time *t*_*f *_- *t*_0_.

The results are given in Figure a showing the percentage of convergence to the global minimum, local minima or failure of Box 5, Box 10, and Box 100, respectively. In the rather artificial case of zero noise shown in Figure a multiple-shooting performs reasonably well while already a significant fraction of the single shooting trials converge to a local mimimum. Figure b presents the results obtained using data with 10% noise to signal ratio. Adding noise deteriorates the performance of both approaches, which can be seen by comparing Figure a and Figure b. As anticipated, multiple-shooting outperforms single shooting, since it reduces the multimodality of the problem. However, multiple-shooting tends to fail more often than single-shooting for large box sizes. Even for this rather simple example the chance of getting trapped in a local solution or to fail is quite significant and increases with increasing noise to signal ratio. The corresponding total computational costs for both methods are summarized in Table 1. Since different platforms are used for our study all CPU times are transformed to a Pentium (178 MFlops) using Linpack benchmark tables. Table 1 exemplifies the trade-off between robustness (multiple-shooting) and speed (single shooting).

**Table 1:** Computational costs in the STAT5 case study (in seconds) for 0% and 10% noise to signal ratio, respectively.

Simulated data with 0%/10% noise				
Box Size	SS	MS	SRES	Hybrid

5	65/80	140/155	30/46	9/10
10	86/90	317/453	34/55	10/11
100	141/170	950/1095	58/80	17/22

The CPU time is normalized using the Linpack benchmark table and is in case of the multistarts of the single shooting (SS) and multiple-shooting (MS) method the sum over all restarts. Increased robustness of MS results in substantially higher computational cost compared to SS. The hybrid is about 3–4 times faster than SRES manifesting the advantage of the proposed method.

In contrast to the local methods, both, the global search strategy SRES and the hybrid approach, converged in all cases to the global optimum which emphasises the strength of global methods. Note that results obtained by DE are comparable to SRES and are therefore omitted. The power of the hybrid strategy can be appreciated considering the average computational cost as shown in Table 1. Using the hybrid reduces the computational load significantly by a factor of four. Due to the systematic switching point calculation no further adjustments were necessary to obtain this significant emendation.

### Oscillatory feedback control system: Goodwin's model

Parameter estimation for oscillating systems is usually more involved than for systems showing a transient behavior. A well known model describing oscillations in enzyme kinetics is the model suggested by Goodwin [30]. It consists of the following set of ordinary differential equations:

(9) $$\begin{array}{c} \dot{x}=\frac{a}{A+z^{\sigma }}-bx\\ \dot{y}=\alpha x-\beta y\\ \dot{z}=\gamma y-\delta z. \end{array}$$

Here, *x *represents an enzyme concentration whose rate of synthesis is regulated by feedback control via a metabolite *z*. The intermediate product *y *regulates the synthesis of *z*. Oscillatory behaviour is not a necessary characteristic of this set of equations. Different values for the parameters may result in limit cycle oscillations, damped oscillations or monotonic convergence to a steady state. In fact, only a restricted range of parameter values result in oscillations. The following values have been used here *x*(0) = 0.3617, *y*(0) = 0.9137, *z*(0) = 1.3934, for the initial conditions and *a *= 3.4884, *A *= 2.1500, *b *= 0.0969, *α *= 0.0969, *β *= 0.0581, *γ *= 0.0969, *σ *= 10, and *δ *= 0.0775, for the model parameters, resulting in oscillatory behavior.

As with the previous case the problem is first approached using multistarts where either single shooting or multiple-shooting are employed. The initial guess of each restart is randomly chosen from the intervals [0, 5] (Box 5), [0, 10] (Box 10) and [0, 100] (Box 100), respectively, for both the parameters and initial conditions using a uniform distribution and two values 0% and 10% noise to signal ratio. The results are summarized in Figure showing the percentage of convergence to the global minimum, local minima or failure for different box sizes. Both local methods encounter difficulties in finding the global optimum, single shooting fastly steps in local minimima or diverges and only on a reduced percentage of the runs converges to the global solution, whereas multiple-shooting performs in all cases better than single shooting at the expense of higher computational costs. In case of the global approaches only DE, under the choice of robust thus slower strategy parameters, was able to find the global minimum, whereas no convergent fit was obtained using SRES. This emphasizes the difficulties in finding the optimal solution for oscillatory systems even for global search strategies. Figure (**a**: 0% noise to signal ratio, **b**: 10% noise-to-signal ratio) shows representative convergence curves for the DE and the hybrid to the global optimum of the Goodwin problem given by Eq. (9). The benefit of the hybrid can be appreciated by comparing the left panel (DE) with the right panel (hybrid). For box size 10 the hybrid converges almost ten times faster while for larger box sizes the asset is even more pronounced. This is also reflected by the CPU times presented in Table 2. It is important to note that this advantage has been obtained without costly adjustment of the switching point as a consequence of the systematic switching strategy employed in the proposed hybrid method. Note moreover that the hybrid may use a faster strategy for DE which further enhances efficiency.

**Table 2:** Computational costs in the Goodwin case study (in seconds) for 0% and 10% noise to signal ratio, respectively.

Simulated data with 0%/10% noise				
Box Size	SS	MS	DE	Hybrid

5	213/409	907/1153	108/104	13/12
10	326/423	1340/1443	972/846	16/14
100	453/472	733/1021	1320/1370	30/26

The CPU time is normalized using the Linpack benchmark table and is in case of the multistarts of the single shooting (SS) and multiple-shooting (MS) method the sum over all restarts. As in the case of the STAT5 example the improved robustness of multiple-shooting gives rise to increased computational cost compared to single shooting. The benefit of the hybrid becomes evident by the fact that the computational cost of the hybrid is about 8 times lower than DE for the Box 5, 60 times lower for the Box 10 and around 40 for Box 100.

## Conclusion

In this study we present a new hybrid strategy as a reliable method for solving challenging parameter estimation problems encountered in systems biology. The proposed method presents two advantages over previous hybrid methods: First, it is equipped with a switching strategy which allows the systematic determination of the transition from the local to global search. This avoids computationally expensive tests in advance and constitutes a major benefit of the proposed method. Second, using multiple-shooting as the local search procedure reduces the multi-modality of the non-linear optimization problem. Because multiple-shooting avoids possible spurious solutions in the vicinity of the global optimum it outmatches the initial value approach (single shooting) yielding an enhanced robustness of the hybrid.

We analyzed the performance of this new approach using two examples: the dynamical model of the STAT5 signaling pathway suggested in [29] and the Goodwin model describing oscillating processes [30]. The hybrid was able to converge to the global solution in all runs performed with significant reductions in the computational cost. Moreover a comparison with other search strategies reveals that the hybrid results in a better compromise efficiency-robustness. In conclusion the proposed hybrid provides a robust and convenient method for parameter estimation problems occurring in systems biology.

## Authors' contributions

C.F. initiated the work, M.P. implemented the multiple-shooting algorithm. M.P. and E.B.C. implemented the hybrid algorithm. E.B.C. performed the simulations. E.B.C., C.F., and M.P. drafted the manuscript. J.T. and J.B. proposed the main idea, gave valuable advises and helped to draft the manuscript. All authors read and approved the final manuscript.

**Figure 1:**
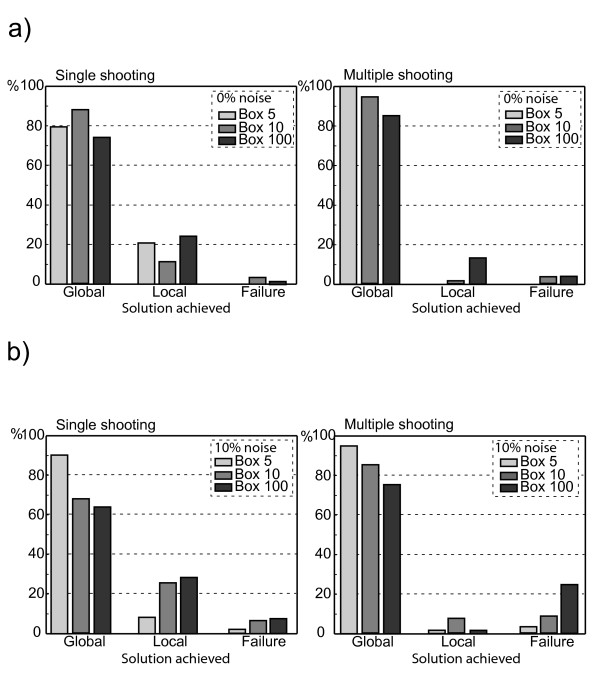
Comparison of the multistart of the generalized-quasi-Newton within single and multiple-shooting for the JAK/STAT5 pathway. Shown is the percentage of convergence to the global minimum, local minima or failure of the optimisation method using 100 restarts. The initial guess of each restart is randomly chosen from interval [0, 5] (Box 5), [0, 10] (Box 10), and [0, 100] (Box 100) using a uniform distribution. **a**) Noise-to-signal ratio is zero. As anticipated, multiple-shooting (right panel) performs better than single shooting (left panel). **b**) Same as in a), but using a noise-to-signal ratio of 10%.

**Figure 2:**
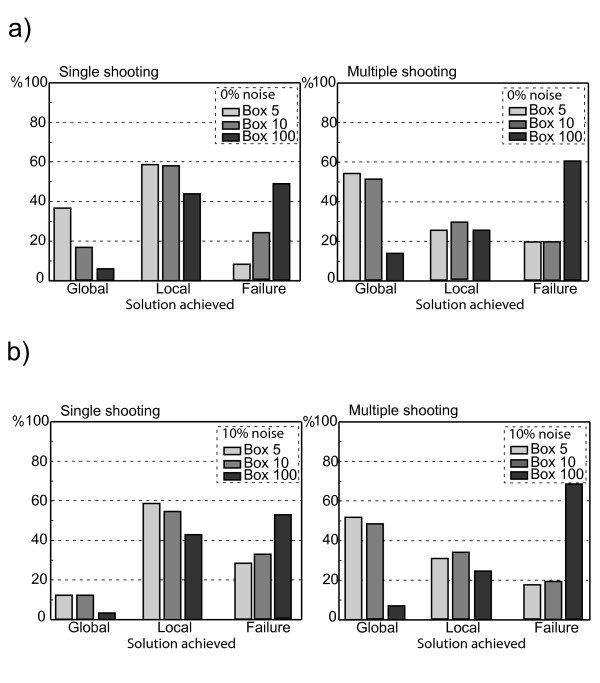
Comparison of the multistart of the generalized-quasi-Newton within single and multiple-shooting for the Goodwin model. Shown is the percentage of convergence to the global minimum, local minima or failure of the optimisation method using 100 restarts. The initial guess of each restart is randomly chosen from interval [0, 100] (Box 100), and [0, 1000] (Box 1000) using a uniform distribution. **a**) Noise-to-signal ratio is zero. **b**) Same as in a), but using a noise-to-signal ratio of 10%.

**Figure 3:**
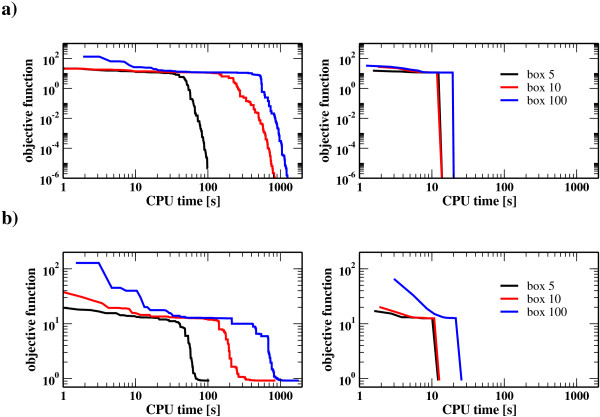
Convergence curves for the DE and the hybrid method to the global optimum of the Goodwin problem given by Eqs. (9). **a**) 0% noise to signal ratio. The left figure shows the value of the objective function as a function of CPU time for different box sizes. CPU time is normalied using the Linpack Benchmark table. The right figure displays the convergence of the hybrid strategy. **b**) Same as in a) but with 10% noise to signal ratio. The difference between the final value of the cost function in **a**) and **b**) is due to the added noise.
